# A SILAC-Based Method for Quantitative Proteomic Analysis of Intestinal Organoids

**DOI:** 10.1038/srep38195

**Published:** 2016-11-30

**Authors:** Alexis Gonneaud, Christine Jones, Naomie Turgeon, Dominique Lévesque, Claude Asselin, François Boudreau, François-Michel Boisvert

**Affiliations:** 1 Department of Anatomy and Cell Biology, Université de Sherbrooke, 3201 Jean-Mignault, Sherbrooke, Québec, J1E 4K8, Canada

## Abstract

Organoids have the potential to bridge 3D cell culture to tissue physiology by providing a model resembling *in vivo* organs. Long-term growing organoids were first isolated from intestinal crypt cells and recreated the renewing intestinal epithelial niche. Since then, this technical breakthrough was applied to many other organs, including prostate, liver, kidney and pancreas. We describe here how to apply a SILAC-based quantitative proteomic approach to measure protein expression changes in intestinal organoids under different experimental conditions. We generated SILAC organoid media that allow organoids to grow and differentiate normally, and confirmed the incorporation of isotopically labelled amino acids. Furthermore, we used a treatment reported to affect organoid differentiation to demonstrate the reproducibility of the quantification using this approach and to validate the identification of proteins that correlate with the inhibition of cellular growth and development. With the combined use of quantitative mass spectrometry, SILAC and organoid culture, we validated this approach and showed that large-scale proteome variations can be measured in an “organ-like” system.

Several methods for quantifying protein changes by mass spectrometry have been developed. While each have their advantages and disadvantages, SILAC (Stable isotope labelling by amino acids in cell culture) remains a strategic choice to analyze simultaneously multiple samples in different conditions^1^. Proteins from samples differentially labelled after metabolic incorporation of isotopic amino acids are pooled before further sample processing, minimizing bias due to handling, and thus increases reproducibility over chemical labelling or label-free quantification approaches^2^. However, SILAC is not applicable to non-cultured samples such as clinical samples, animals and certain organisms requiring complex culture media.

Three-dimensional (3D) cell culture strategies have been developed to better reflect tissue characteristics in both normal and diseased physiological conditions^3^,^4^. Significant progress has been recently made in defining optimal conditions to allow growth, expansion and differentiation of intestinal epithelial stem cells^5^,^6^ as well as several other tissue stem cells^7^,^8^,^9^. Unlike cancer cell lines, organoids maintain all the variables specific to the original epithelial cell, including the tumor cell^10^. As a result, organoid culture is becoming the preferred strategy in personalized medicine, as it allows the testing of existing and experimental treatments on samples with distinct genomic individual signatures^11^,^12^. The ability to culture these mini-organs raises the question of their use for isotope incorporation to perform SILAC based quantitative proteomics. If successful, this would allow accurate protein quantification in a physiologically relevant system (Fig. 1).

To address the feasibility of a proteomic approach on organoids, there was a need to determine the extent of contamination from Matrigel proteins, which are needed for organoid growth. Indeed, Matrigel proteins could mask the proteins isolated from the embedded biological material^13^. Organoids were isolated from Matrigel with the non-enzymatic MatriSperse dissociation method. Several washes with cold PBS promoted the removal of Matrigel and the isolation of intact organoids, prior to solubilisation of proteins and in-solution tryptic digestion, LC-MS/MS analysis and protein identification. Results showed that while Matrigel proteins, such as collagens and laminins, were distinguished, over 2500 proteins were identified in these purified organoids, demonstrating that the presence of Matrigel did not hinder the identification of proteins from organoids (Supplementary Table 1).

In order to perform SILAC quantification, near complete isotope incorporation must be achieved in the growth media. It is thus essential that all components of the culture media provide the correct isotope with no contribution from other isotopic amino acids. To create a ‘SILAC organoid’ media, R-spondin 1- or Noggin-expressing 293T cells^14^ were grown in three different SILAC media containing arginine and lysine, either with normal isotopes of carbon and nitrogen (i.e. ^12^C^14^N, light), L-arginine-^13^C_6_^14^N_4_ and L-lysine-^2^H_4_ (medium) or L-arginine-^13^C_6_-^15^N_4_ and L-lysine-^13^C_6_-^15^N_2_ (heavy). Conditioned media were harvested, filtered and mixed to a final contribution of 20% of R-spondin 1 conditioned medium, 10% of Noggin conditioned medium and 70% of Advanced DMEM/F-12 Flex medium supplemented with the same isotopic amino acids. Organoids were cultured in these light, medium or heavy SILAC organoid media for several passages performed at approximately every 5 to 7 days of culture. Organoids were harvested at 10, 15, 20, 25 and 30 days of culture to establish an incorporation curve of SILAC isotopes (Fig. 2a). Organoids isolated from different media at different times were mixed 1:1:1 and trypsin-digested prior to mass spectrometry analysis (Fig. 2a and Supplementary Table 2). We observed an increase in isotope incorporation until the curve reached a plateau at >90% of incorporation, for both heavy and medium isotopes (Fig. 2a). The data indicate that nearly complete SILAC incorporation in organoids requires approximately 20 days of incorporation (Fig. 2a), which corresponds to four passages in SILAC organoid media.

We then determined whether this process could be accelerated by using frozen organoids which already had completed SILAC incorporation. Organoids were grown in the different SILAC media for 20 days, then frozen in SILAC conservation media containing dialyzed serum, DMSO and the desired SILAC Advanced DMEM/F-12 Flex media for long term storage in liquid nitrogen. After two weeks, organoids were thawed and re-introduced immediately in SILAC media to assess organoid growth for 10 days. Organoids appeared normal, without any changes in growth. Proteins were then analyzed by mass spectrometry to determine the incorporation ratios, which still indicated an enrichment of >90%, as above (data not shown).

To determine whether protein expression changes could be quantified in SILAC-treated organoid cultures, we determined the effect of CI994, a class I HDAC inhibitor for Hdac1, 2 and 3^15^. HDAC inhibitors have been shown to affect intestinal epithelial cell growth and differentiation in intestinal organoid cultures^5^. Organoids were treated during 5 days with either DMSO or 5 μM of CI994. Macroscopically, CI994-treated organoids were smaller, with decreased crypt-budding formation, indicating potential changes in the coordination of HDAC-dependent mechanisms controlling self-renewal and differentiation of the intestinal epithelium (Fig. 2b). To determine reproducibility, two replicates from the initial organoid culture, including sample preparation and protein identification by mass spectrometry, were performed. A Pearson coefficient of 0.85 was measured for the treatment with CI994, indicating a very strong reproducibility when comparing populations of organoids (Fig. 2c and Supplementary Table 3). This allowed the identification of proteins that were consistently upregulated or downregulated following CI994 treatment.

To confirm the physiological relevance of protein expression changes, we characterized the proteins with the largest increase or decrease following treatment with CI994 (Fig. 2d). Gene ontology analysis of the biological processes, enriched in the proteins increased by more than 50% using DAVID^16^, identified processes mainly associated with metabolic pathways such as oxydoreduction (p-value of 29e-19) and carbohydrate catabolic process (p-value of 6.60e-10) as well as several processes implicated in the metabolism and absorption of complex sugars (Supplementary Table 4 and Fig. 3a). For proteins decreased by 50%, associations with DNA replication initiation (p-value of 1.30e-7), cell cycle regulation (p-value of 6.0e-5), DNA packaging (p-value of 8.3e-7) and chromosome organization (p-value of 6.3e-6) were consistent with the observation that organoids were smaller and that proliferating crypt-budding structures were less developed after 5 days of treatment with the HDAC inhibitor (Supplementary Table 4 and Fig. 3a). In addition, altered enterocyte differentiation was observed in CI994-treated organoids. For example, antimicrobial Reg3β and Reg3γ proteins, as well as sucrase-isomaltase (Sis) and intestinal alkaline phosphatase, were increased. In contrast, goblet (Muc2) and endocrine (ChgB and GIP) proteins were reduced (Fig. 2c). This was consistent with the findings that wide inhibition of HDAC activities reduces secretory cell lineage differentiation^5^.

These observations were confirmed by quantitative PCR for differentially expressed proteins after CI994 treatment. For example, ChgB and Muc2 mRNA expression was decreased in CI994-treated organoids, whereas Reg3β and Sis mRNA expression was increased (Fig. 3b). Moreover, Reg3β and Sis protein levels were increased, as determined by Western blot analysis, thus confirming the proteomic data showing an increase in Reg3β and Sis proteins in CI994-treated organoids (Fig. 3c and Supplementary Table 4). Interestingly, mRNA levels of the Lgr5 stem cell marker were decreased following CI994 treatment (Fig. 3b), suggesting reduced numbers of stem cells and correlating with the smaller and less developed aspect of organoids observed. Interestingly, this effect on Lgr5 expression is different from VPA treatment^5^. This could be explained by different HDAC inhibitory properties of both compounds. Both VPA and CI994 are considered class I HDAC inhibitors, with little activity on class II HDAC. While VPA inhibits more efficiently Hdac1 and Hdac2 rather than Hdac3, CI994 targets similarly Hdac1, Hdac2 and Hdac3^15^,^17^. Thus, this suggests that residual class I HDAC activity, through Hdac3, may be necessary to maintain Lgr5-expressing cell pools. However, inhibition of the three Hdac isoforms with CI994 could lead to growth arrest and subsequent organoid growth defects.

Our data demonstrate the feasibility of using SILAC for quantitative proteomic studies with organoids. We describe a culture method to ensure >90% isotopic incorporation. This methodology allows the quantification of changes at the proteome level in 3D cell cultures during cellular differentiation, or in response to different inhibitors and drugs. With the recent promise of adapting organoid technology for precision medicine^18^, this new methodology will become advantageous to quantify global changes in protein expression, thereby identifying novel signalling pathways and targets upon defined environmental context.

## Methods

### Production of SILAC Noggin and R-spondin 1 conditioned media

Noggin-Fc and R-spondin 1-Fc were produced using 293T cell lines stably expressing R-spondin 1-Fc (kindly provided by Dr. C. Kuo, Stanford University, USA) or Noggin-Fc (kindly provided by Dr. G.R. van den Brink, Hubrecht Institute, The Netherlands). These cells were cultured in T75 cm^2^ flasks with DMEM (without arginine or lysine) medium supplemented with 10% FBS, GlutaMAX (1X), HEPES (1X), penicillin (100 U/mL) and streptomycin (100 μg/mL). Arginine and lysine were added in either light (Arg0, Sigma, A5006; Lys0, Sigma, L5501), medium (Arg6, Cambridge Isotope Lab (CIL), CNM-2265; Lys4, CIL, DLM-2640), or heavy (Arg10, CIL, CNLM-539; Lys8, CIL, CNLM-291) forms to final concentrations of 28 μg/ml for arginine and 49 μg/ml for lysine. Selective antibiotic puromycin (10 mg/mL) for Noggin-Fc production or Zeocin (10 mg/mL) for R-spondin 1-Fc production were added in the media during cell passages. Selection was stopped when cells were amplified in T175 cm^2^ flasks. At confluence, cells were amplified in Advanced DMEM/F12 medium (without arginine or lysine) and supplemented with GlutaMAX (1X), HEPES (1X), penicillin (100 U/mL) and streptomycin (100 μg/mL) for 7 days, as well as the appropriate SILAC isotopes. Medium was then removed and centrifuged at 1000 rpm for 5 min. Supernatants containing R-spondin 1-Fc or Noggin-Fc were filtered through a 0.22 μM membrane and stored at −80 °C. Expression of R-spondin or Noggin was measured by dot blots through the Fc using secondary antibodies coupled to HRP.

### Organoid culture

Two-month-old mice were used for intestinal epithelial cell isolation. For each culture, crypts were isolated from one mouse jejunum with EDTA. Animal investigations and the experimental protocol were approved by the Institutional Animal Research Review Committee of the Université de Sherbrooke (protocol 360-14B), and all methods were carried out in accordance with regulations. Briefly, jejunum was opened longitudinally and rinsed in a Petri dish with cold PBS 1X. The intestine was then cut into pieces of 5 mm in length and fragments washed by stirring in a 50 mL Falcon tube containing PBS 1X. Intestinal fragments were transferred in a 50 mL Falcon tube containing 20 mL of 30 mM EDTA and incubated for 5 min on ice with gentle stirring. Fresh EDTA was substituted and further incubation was done for 20 min on ice. EDTA was replaced with 40 mL of PBS 1X. The Falcon tube was shaken vigorously for 5–8 min or until dissociated crypts were visualized in the solution as determined under a microscope. The solution was filtered using a 70 μm cell strainer with crypts going through as opposed to residual villi being retained by the filter. Crypts were then centrifuged at 150× g for 5 min. The cell pellet was washed twice with 25 mL of Advanced DMEM/F-12 medium. A second centrifugation was performed at 150× g for 5 min. The pellet was then resuspended in 1 mL of Matrigel (BD Corning) and plated in a 48-well plate (Corning Costar) (20 μL/well).

### Organoid SILAC media

The organoid SILAC media added to Matrigel embedded cells consisted of 70% SILAC Advanced DMEM/F-12 Flex media (ThermoFisher) supplemented with 1,25 mM N-acetylcysteine (Sigma), 50 ng/mL EGF (Life Technologies), B27 supplement 1X (Life Technologies), N2 supplement 1X (Life Technologies), 10% SILAC Advanced DMEM/F-12 Noggin media, and 20% SILAC Advanced DMEM/F-12 R-Spondin 1. All culture medium contained arginine and lysine, either with the normal light isotopes of carbon, hydrogen and nitrogen (*i.e.*^12^C^14^N) (light – “L”), or else with L-arginine-^13^C_6_^14^N_4_ and L-lysine-^2^H_4_ (medium – “M”) or with medium containing L-arginine- ^13^C_6_-^15^N_4_ and L-lysine-^13^C_6_-^15^N_2_ (heavy – “H”). In some experiments, organoids were treated during 5 days with 5 μM CI994 (Cayman Chemical), a class I HDAC inhibitor. CI994 was diluted in DMSO and control organoids were supplemented with DMSO. We chose a CI994 concentration resulting in the most apparent phenotypic effect on organoid growth and appearance, in order to validate our method.

### Incorporation assay

Organoids (average of 250 organoids/well) were maintained in SILAC organoid light, medium or heavy media for several passages (every 5 to 7 days of culture) to allow SILAC isotope incorporation. Samples were collected at different time points (10, 15, 20, 25 and 30 days of culture) and analyzed by mass spectrometry to estimate incorporation by using medium/light and heavy/light ratios.

### Conservation and re-culturing assay

Organoids grown in SILAC organoid light, medium or heavy media for 20 days were harvested and resuspended in SILAC conservation media composed of 70% dialyzed fetal bovine serum (Invitrogen), 15% DMSO and the desired SILAC Advanced DMEM/F-12 Flex light, medium or heavy media, stored at −80 °C overnight before being transferred in liquid nitrogen. After 2 weeks, frozen organoids were thawed and grown as described before in the different SILAC media for 10 days (technically for a total of 30 days). Organoids were collected and analysed by mass spectrometry to confirm isotope incorporation.

### Sample collection

To assess level of isotopic incorporation and variability among CI994 treatments, organoids were processed for HPLC-MS/MS analysis. The media was removed and each well was washed with cold PBS 1X. 300 μL of MatriSperse (Corning) was added per well and incubated for 30 min at 4 °C. Organoids were then collected in Protein LoBind tube (Eppendorf) and centrifuged at 7000 rpm for 3 min at 4 °C. The supernatant was removed and the cells were washed three times with cold PBS 1X, by centrifugation at 7000 rpm for 3 min at 4 °C. Cell pellets were then frozen at −80 °C prior to mass spectrometry preparation and analysis.

### In-solution digestion

Frozen organoid samples were resuspended in a fresh 10 mM HEPES (pH 7.0–7.6), 8 M urea solution. Proteins were reduced in 3.24 mM dithiothreitol (DTT) and alkylated in 13.5 mM iodoacetamide. The final concentration of urea was lowered to 2 M with the addition of 50 mM ammonium bicarbonate (NH_4_HCO_3_). After BCA dosage, 15 μg of light and heavy condition samples were pooled and digested by trypsin prior to mass spectrometry analysis.

### Gel electrophoresis and in-gel digestion

For each time point, proteins were reduced in 10 mM dithiothreitol (DTT), alkylated in 50 mM iodoacetamide prior to boiling in loading buffer, then separated by one-dimensional SDS-PAGE (4–12% Bis-Tris Novex mini-gel, Invitrogen) and visualized by colloidal Coomassie staining (SimplyBlue Safestain, Invitrogen). The entire protein gel lanes were excised and cut into 8 slices each. Every gel slice was subjected to in-gel digestion with trypsin^19^. The resulting tryptic peptides were extracted by 1% formic acid, then 100% acetonitrile, lyophilized in a speedvac, and resuspended in 1% formic acid.

### Liquid chromatography-tandem MS (LC-MS/MS)

Trypsin digested peptides were separated using an Ultimate U3000 (Dionex Corporation) nanoflow LC-system. Ten microliters of sample (a total of 2 μg peptide) were loaded with a constant flow of 4 μl/min onto a PepMap C18 trap column (0.3 mm id × 5 mm, Dionex Corporation). Peptides were then eluted onto a PepMap C18 nano column (75 μm × 50 cm, Dionex Corporation) with a linear gradient of 5–35% solvent B (90% acetonitrile with 0.1% formic acid) over 240 min with a constant flow of 200 nl/min. The performance liquid chromatography (HPLC) system was coupled to an OrbiTrap Q Exactive, via an EasySpray source. The spray voltage was set to 1.5 kV and the temperature of the column was set to 40 °C. Full scan MS survey spectra (*m*/*z* 350–1800) in profile mode were acquired in the Orbitrap with a resolution of 70,000 after accumulation of 1,000,000 ions. The ten most intense peptide ions were fragmented by collision-induced dissociation (normalized collision energy 35% with a resolution of 17,500) after the accumulation of 50,000 ions. Maximal filling times were 250 ms for the full scans and 60 ms for the MS/MS scans. Precursor ion charge state screening was enabled and all unassigned charge states as well as singly, 7 and 8 charged species were rejected. The dynamic exclusion list was restricted to a maximum of 500 entries with a maximum retention period of 60 s and a relative mass window of 10 ppm. The lock mass option was enabled for survey scans to improve mass accuracy. The lock mass option was enabled and data were acquired using the XCalibur software.

### Quantification and bioinformatic analysis

Protein identification and SILAC quantification were performed with MaxQuant version 1.5.2.8^20^ using the Uniprot mouse protein database containing 89,422 proteins, to which 175 commonly observed contaminants and all the reversed sequences had been added. The initial mass tolerance was set to 7 p.p.m. and MS/MS mass tolerance was 0.5 Da. Enzyme was set to Trypsin/P with 2 missed cleavages. Carbamidomethylation of cysteine was searched as a fixed modification, whereas *N*-acetyl protein and oxidation of methionine were searched as variable modifications. Identification was set to a false discovery rate of 1%. To achieve reliable identifications, all proteins were accepted based on the criteria that the number of forward hits in the database was at least 100-fold higher than the number of reverse database hits, thus resulting in a false discovery rate of less than 1%. A minimum of 3 peptides were quantified for each protein, and quantification performed only in the presence of 3 ratio counts. The mass spectrometry data have been deposited to the ProteomeXchange Consortium (http://proteomecentral.proteomexchange.org) via the PRIDE partner repository with the dataset identifier PXD004607.

### QPCR analysis

2 ng of cDNAs, prepared from CI994-treated and untreated organoid mRNAs, were used for qPCR using the RT2 SYBR Green ROX qPCR Master Mix (Qiagen), and Up and Down oligonucleotides specific to ChgB, Muc2, Reg3β, Lgr5 and Sis were used for amplification (Supplementary Table 5). Relative quantification was assessed by Porphobilinogen deaminase (Pbgd) amplification. Ten min incubation at 95 °C was followed by 40 cycles of 10 s at 95 °C, 10 s at 60 °C and 20 s at 72 °C. Results are expressed as the mean ± S.E.M. Statistical significance was determined by performing unpaired t-tests.

### Western blot analysis

Whole organoid lysates were prepared with a lysis buffer containing 8 M urea. 15 μg of proteins from each CI994-treated and untreated condition were separated on 4–12% Bis-Tris NuPage precast gels (Life Technologies) and transferred to PVDF blotting membranes (Roche Applied Science). Western blotting was performed as described previously^21^. Antibodies used were: goat anti-Reg3β (AF1996, R&D Systems), goat anti-SIS (sc-27603, Santa Cruz Biotechnology), mouse anti-actin (MAB-1501, EMD Millipore), donkey anti-goat (sc-2020, Santa Cruz Biotechnology) and goat anti-mouse (sc-2005, Santa Cruz Biotechnology). Immune complexes were detected with Amersham ECL™ Western blotting detection reagents (GE Healthcare), according to the manufacturer’s instructions.

### Statistical analysis

**Figure 2:**
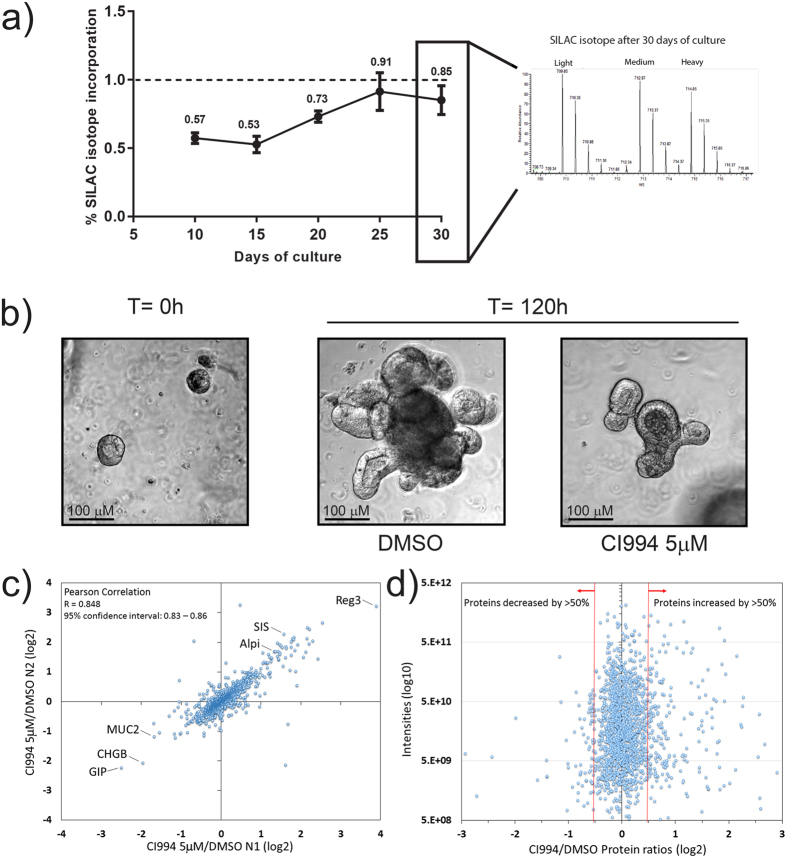
Incorporation of SILAC isotopes in organoid cultures and effect of the class I HDAC inhibitor CI994 on organoid growth and development. (**a**) Organoids were grown continuously in SILAC medium containing arginine and lysine with light isotopes of carbon, hydrogen and nitrogen (i.e. ^12^C^14^N) (light), or with medium containing L-arginine-^13^C_6_-^15^N_4_ and L-lysine-^13^C_6_-^15^N_2_ (heavy). Intestinal organoids were amplified and split every five days. From days 10 to 30, levels of isotope incorporation were evaluated by mass spectrometry analysis by combining equal amounts of organoid lysates prior to sample processing, protein identification and quantification. (**b**) Micrograph of intestinal organoids before and after 5-day treatment with either DMSO or CI994 at 5 μM. Scale bars represent 100 μm. (**c**) Evaluation of reproducibility between the different assays: ratios from one experimental repeat (N1) CI994 5 μM/DMSO (x axis) versus the second repeat (N2) CI994 5 μM/DMSO (y axis) for identified proteins. A correlation between N1 and N2 CI994 5 μM/DMSO experiments was observed with Pearson correlation (*R* = 0.848).(**d**) The average CI994/DMSO ratios of proteins over the average peptide intensities, to identify proteins with an increase or decrease in expression following treatment with the HDAC inhibitor CI994.

**Figure 3:**
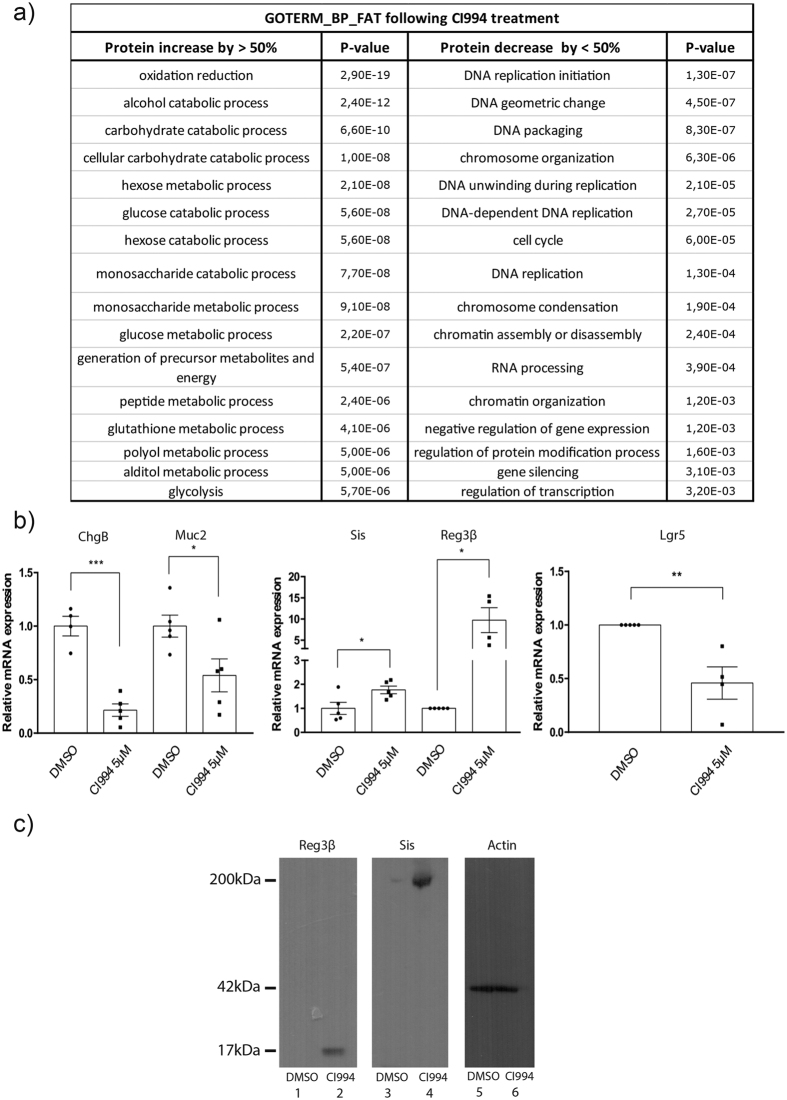
CI994 treatment affects organoid differentiation and development processes. (**a**) Gene ontology term enrichment of biological processes for decreased and induced proteins (<50% and >50%) in CI994-treated organoids. Gene ontology analysis was done using the EASE Score from DAVID 2.0 (p-value ≤ 0.05). (**b**) Total RNAs were isolated from organoids treated with or without CI994. Expression levels of selected genes, namely ChgB, Muc2, Reg3β, Sis and Lgr5 were measured by qPCR in comparison to PBGD expression as a control. Results represent the mean ± S.E.M. (*P < 0.05). Statistical significance was determined by unpaired t tests. (**c**) Reg3β and Sis protein expression following CI994 treatment was monitored by Western blot analysis, with actin as a protein loading control.

Pearson correlation coefficients were measured to evaluate the correlation between the two repeats of the CI994 5 μM/DMSO experiments (Fig. 2c). Gene ontology analysis was done using the EASE score (a modified Fisher Exact P-Value) from DAVID 2.0 (p-value ≤ 0.05) (Fig. 3a). Results of qPCR analysis are expressed as the mean ± S.E.M. and statistical significance was determined using an unpaired t tests (Fig. 3b).

## Additional Information

**How to cite this article**: Gonneaud, A. *et al*. A SILAC-based method for quantitative proteomic analysis of intestinal organoids. *Sci. Rep.* **6**, 38195; doi: 10.1038/srep38195 (2016).

**Publisher's note:** Springer Nature remains neutral with regard to jurisdictional claims in published maps and institutional affiliations.

## Supplementary Material

Supplementary Information

Supplementary Table 1

Supplementary Table 2

Supplementary Table 3

Supplementary Table 4

Supplementary Table 5

## Figures and Tables

**Figure 1:**
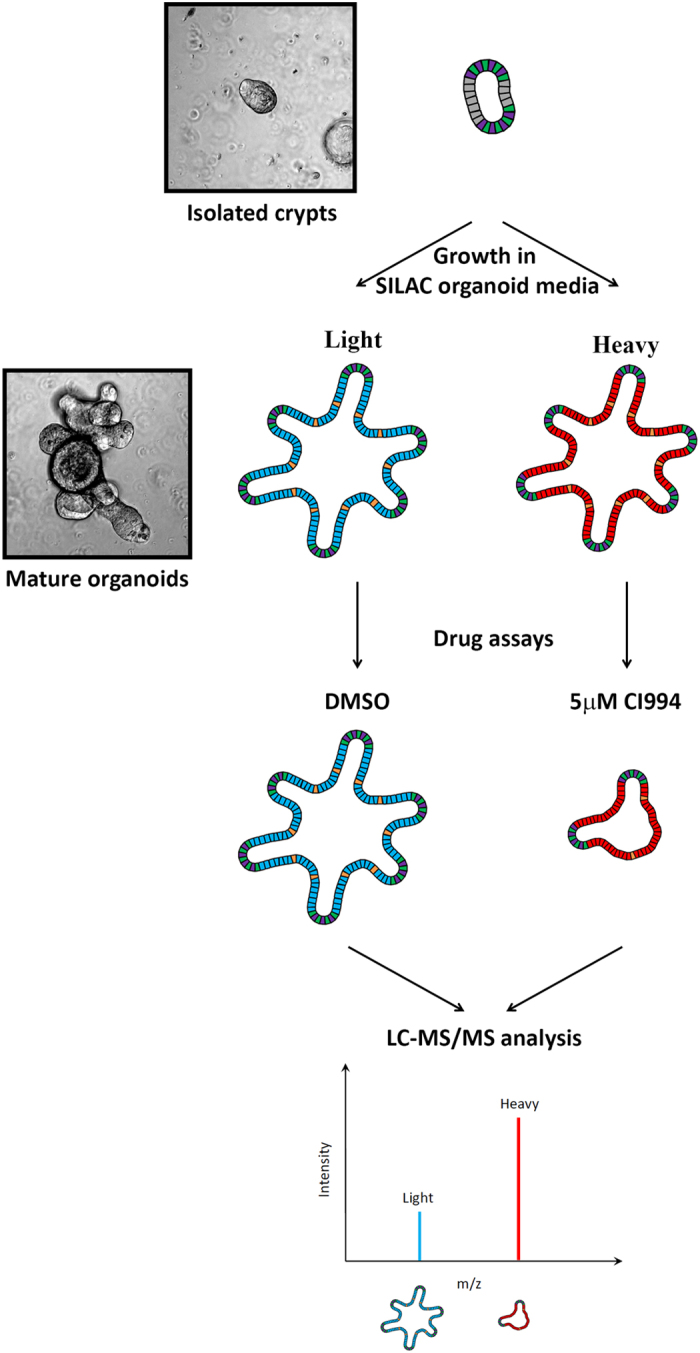
Experimental protocol of SILAC labelling of organoids and mass spectroscopy analysis. Outline of the SILAC mass spectrometry experimental design for organoids. Intestinal organoids from isolated crypts were grown in different SILAC media for 20 days. Different drugs or treatment can then be used and SILAC allows quantification of changes in protein expression. The effect of a class I HDAC inhibitor, CI994, was used to validate the feasibility of this approach.
