# Supplementary material for: A SILAC-Based Method for Quantitative Proteomic Analysis of Intestinal Organoids
Source: Sci Rep. 2016 Nov 30;6:38195. doi: 10.1038/srep38195 (PMC5128881; doi:10.1038/srep38195)
Supplement: Supplementary Information [file srep38195-s1.pdf]

A SILAC-Based Method for Quantitative Proteomic Analysis of Intestinal Organoids

Alexis Gonneaud, Christine Jones, Naomie Turgeon, Dominique Levesque, Claude Asselin, François Boudreau, François-Michel Boisvert

SUPPLEMENTARY TABLES

The following tables provide the data used to generate the graphs from figure 2:

Supplementary Table 1 - Mass spectrometry analysis of organoids isolated from Matrigel.

Supplementary Table 2 - Mass spectrometry analysis of SILAC label incorporation.

Supplementary Table 3 – Reproducibility data for CI994 treated organoids.

Supplementary Table 4 - Mass spectrometry analysis of organoids treated with HDAC inhibitor CI994.

Supplementary Table 5 – Sequences of oligonucleotides used for qPCR.
